# Stress Testing the Capacity of Health Systems to Manage Climate Change-Related Shocks and Stresses

**DOI:** 10.3390/ijerph15112370

**Published:** 2018-10-26

**Authors:** Kristie L. Ebi, Peter Berry, Katie Hayes, Christopher Boyer, Samuel Sellers, Paddy M. Enright, Jeremy J. Hess

**Affiliations:** 1 Center for Health and the Global Environment, University of Washington, 4225 Roosevelt Way NE Suite 100, Seattle, WA 98105, USA; cboyer10@uw.edu (C.B.); sellers1@uw.edu (S.S.); jjhess@uw.edu (J.J.H.); 2 Department of Environmental and Occupational Health Sciences, School of Public Health, University of Washington, Seattle, WA 98105, USA; 3 Department of Global Health, Schools of Medicine and Public Health, University of Washington, Seattle, WA 98105, USA; 4 Climate Change and Innovation Bureau, Health Canada, 269 Laurier Ave. West, Ottawa, ON K1A 0K9, Canada; Peter.Berry@Canada.ca (P.B.); Paddy.Enright@Canada.ca (P.M.E.); 5 Department of Geography and Environmental Management, University of Waterloo, 200 University Avenue W, Waterloo, ON N2L 3G1, Canada; pberry@uwaterloo.ca (P.B.); pmenright@uwaterloo.ca (P.M.E.); 6 Dalla Lana School of Public Health, University of Toronto, 155 College St, Toronto, ON M5T 3M7, Canada; katie.hayes@mail.utoronto.ca; 7 Department of Emergency Medicine, School of Medicine, University of Washington, Seattle, WA 98105, USA

**Keywords:** climate change, climate variability, health risks of climate change, health systems, health workforce, stress test, vulnerability and adaptation assessments

## Abstract

Vulnerability and adaptation assessments can provide valuable input to foster climate-resilient health systems. However, these assessments often do not explore the potential health risks of climate change far outside the range of recent experience with extreme weather events and other climate-related hazards. Climate and health stress tests are designed to increase the capacity of health systems and related sectors to manage potentially disruptive climate-related shocks and stresses. Stress tests focus on hypothetical scenarios, during which it would be difficult for the health system to maintain its essential function of providing services to protect population health. The stress test explores approaches to effectively manage acute and chronic climate-related events and conditions that could directly impact health systems, and climate-related events in non-health sectors that can indirectly impact health outcomes and/or health system function. We provide detailed methods and guidance for conducting climate and health stress tests, centering on three primary activities: (1) preparing and scoping the stress test; (2) successfully conducting the stress test; and (3) communicating the results to key stakeholders to facilitate policy and programmatic reforms.

## 1. Introduction

Climate and other global environmental changes are fundamentally altering our future. Possible futures include a “hothouse earth” if self-reinforcing feedbacks result in continued warming, despite reductions in greenhouse gas emissions [1]. Even stabilizing greenhouse gas emissions to achieve the Paris Agreement goal of limiting warming to 1.5 °C above pre-industrial temperatures is projected to result in a significantly warmer future within the next few decades [2]. Climate variability and change, including increasing temperatures, alterations in the hydrologic cycle, and increases in the frequency and severity of extreme weather and climate events, are associated with a range of adverse health consequences. Impacts can be direct, such as illnesses and deaths from heatwaves and severe storms; or can arise from indirect pathways, such as poor health outcomes associated with human migration, ecosystem changes (e.g., vector-borne diseases), food insecurity, and economic decline [3,4,5]. Some impacts of climate change, such as effects on mental health (e.g., post-traumatic stress disorder, anxiety, depression, violence, suicide) may occur in the short-term or extend over several years, with associated implications for their management by health systems [5].

In addition to these climatic shifts, demographic changes (e.g., aging, migration), urbanization, land use change, and other social determinants of health, will also change, with potential implications for vulnerability to climate-related hazards [6]. All these changes will interact, with possible amplifications of the challenges that individuals, communities, and health systems are projected to face. Health systems include all facets of healthcare (e.g., acute care, chronic care, long-term care, and mental healthcare), as well as all aspects of care delivery (e.g., workforce, care facilities, supply chains, and pharmaceuticals), health governance and leadership, and health information and technology, including surveillance and financing [7].

The focus of health adaptation to climate change has been on strengthening health systems to better manage its impacts. The WHO *Operational Framework for Building Climate Resilient Health Systems* describes the possible implications of climate change for the six building blocks of health systems: leadership and governance, health workforce, health information systems, essential medical products and technologies, service delivery, and climate and health financing [7]. This framework was used, for example, to inform toolkits designed in the United States [8] and Canada [9], and by the Pan American Health Organization [10] to increase the sustainability and resiliency of healthcare facilities in the Americas [11]. While incremental improvements to modify current programs are important first steps to increase resilience, they may lead to inadequate preparation of health systems and overconfidence in their ability to manage multiple concurrent and synergistic climate-related exposures outside the range of historic experiences, as expected with climate change.

These challenges are compounded for health systems struggling to manage the current health risks of climate variability and change due to limited adaptive capacity [3], such as those in many low- and middle-income countries. The Solomon Islands provides an example to illustrate the potentially severe climate-related impacts and challenges for health systems. In that country, many health facilities, including the only tertiary hospital in the country, are located on the seashore, with limited seawall protection and at high risk of storm surges, flooding, and sea level rise [12]. A dengue outbreak in 2013, and flash flooding in 2014, illustrated the extreme weakness and vulnerability of the health system and health facilities due to structural damage and increased patient demand. Following the flooding event, 31 acute and subacute deaths were recorded, as well as a diarrhea outbreak that affected 8584 people. This resulted in a surge of inpatients that strained healthcare staff and depleted medical supplies. Damage to the hospital and surrounding clinics, serving 26,000 people, led to the evacuation of patients [13]. Without major adaptation measures—and, eventually, relocation—the majority of facilities in the Solomon Islands will be inundated in the coming decades [14].

The extended timeframe associated with climate change necessitates a broader scope for risk assessment and risk reduction efforts, increasing the time horizons for decisions. This results in other dynamics within and outside the health sector becoming increasingly important. Health sector capacity, particularly for highly vulnerable populations, will depend on poverty rates [15], healthcare financing [16], and other policy choices [17]. Vulnerabilities will shift because of changes in climate as well as migration, urban form, technology, and access to safe water and improved sanitation, among other development trends [6,18].

To increase resilience to a relatively unexplored aspect of climate-related health risks—increases in shocks and stresses with additional climate change—we propose conducting climate and health stress tests, in conjunction with or following a vulnerability and adaptation assessment to identify and prioritize interventions that promote climate-smart health systems and increase resilience. 

## 2. Some Limitations of Vulnerability and Adaptation Assessments

Climate change and health vulnerability and adaptation (V&A) assessments are designed to provide insights into current and projected future risks of climate variability and change, as well as options for preparing for and effectively managing these risks [19]. Numerous health vulnerability and adaptation assessments have been completed (e.g., [5,20,21]), with many more underway. Since climate change and health is a relatively new issue for ministries and departments of health, and due to limited human and financial resources for conducting V&A assessments and short planning cycles, these assessments tend to focus on reducing vulnerability to current climate variability for a limited number of climate-sensitive health outcomes. In response to these assessments, policies and measures are being implemented to enhance local to national level preparedness within, for example, vector-borne disease control programs.

V&A assessments are rarely comprehensive; a wide range of adverse health outcomes that are expected to be exacerbated by the consequences of climate change are frequently not considered, such as increases in mental health disorders or the population health impacts of migration [22]. The importance of considering mental health in assessments was illustrated by the lingering mental health effects of the 2016 and 2017 western Canada wildfires that prompted the Canadian Mental Health Association to create telephone help lines for people experiencing trauma and distress due to the fires [23]. This support was particularly needed when British Columbia declared a state of emergency as hundreds of wildfires raged throughout the province in summer 2018 [24]. The 2016 US climate change and health assessment [5] included a chapter on mental health, and the next Canadian assessment will include information on this issue.

Another limitation of V&A assessments is that guidance is not designed to identify climate shocks and stresses outside the current range of experience that, alone or in combination with other pressures, could undermine the effectiveness of health system functioning and impact health outcomes. Existing guidance focuses on investigating the effectiveness, strengths, and weaknesses of policies and programs aimed at reducing health risks from current climate variability and recent climate change [19]. V&A guidance does not include evaluation of the day-to-day challenges health systems may face in realistic, but difficult to project, scenarios in an era absent of climactic stationarity. Further, tipping points resulting from cumulative and/or compounding impacts are rarely considered. A climate and health stress test provides an avenue to explore, in detail, what climate change shocks or stresses may mean for the operational capacity of health systems. 

## 3. Climate-Related Shocks and Stresses

Health systems regularly prepare for shocks and stresses (a shock is a single unpredictable event and a stress is an ongoing hardship) not related to climate change, such as pandemics, chronic human resource challenges, demographic changes (e.g., population aging), financial crises, and others. Preparation for stresses tends to be distinct from preparation for shocks, because stresses tend not to compromise function to the point that health systems functionality declines, although, over time, the accumulated effects of stresses can make health systems more vulnerable to shocks. Tipping points have been identified at which health systems begin to exhibit declining performance under standard conditions, highlighting thresholds beyond which operational stresses undermine essential functions [25]. By contrast, shocks necessitate a short-term reorientation of health systems to optimize population health outcomes, rather than their usual focus (in care delivery settings) on optimizing outcomes for individual patients [26].

Shocks and stresses differ in the burdens they impose relative to a system’s resilience and integrity, with shocks generally more intense and short-lived. Examples of shocks include environmental, climatic, or other types of events, such as floods, high winds, landslides, heatwaves or droughts, that can impact health, damage critical public health infrastructure, and impede health system functioning. By contrast, stresses can be beneficial to health systems through hormesis, which primes a system to respond effectively to stress, or be damaging, depending on the net effects. Stresses are characterized by continuous and compounding changes in environmental, climatic, or other types of factors (e.g., increasing desertification, multi-year declines in agricultural production, or chronic economic decline). Shocks and stresses affect population health and health systems through myriad pathways, including direct losses of lives, livelihoods, and infrastructure, and diverting funds from investments in social and economic development to, for example, emergency relief and reconstruction.

The impacts of a shock or stress depend on the magnitude of the hazard, as well as the extent of exposure of human and natural systems to the hazard, the susceptibility of those systems to harm, and their ability to cope with and recover from exposure [27,28]. The record 2017 wildfire season in British Columbia resulted in the temporary closure of 19 health facilities or sites, 880 patients evacuated from facilities, over 700 health services staff displaced and $2.7 million in costs to Interior Health Services for the response [29]. Shocks and stresses can also result in synergistic impacts that reverberate throughout health and other key socioeconomic systems [30,31,32,33,34]. For example, persistent drought and food insecurity in Syria, coupled with social-political and religious tensions, resulted in civil conflict, mass displacement, and a public health crisis [35].

A further challenge is that many health systems operate with little to no surge capacity, which is problematic in the context of growing risks from climate-related shocks and stresses, in both low- and high-resource settings. For example, following Tropical Cyclone Pam in Vanuatu in 2015, the destruction of healthcare infrastructure, combined with low numbers of healthcare personnel and difficulties mobilizing resources and funds, significantly impacted the capacity of health services to deliver curative and preventative services [36,37]. Similarly, addressing mental health after climate-related disasters can be very challenging for some health systems [38]. After Superstorm Sandy struck New York in 2012, the lack of access to care increased the odds of post-traumatic stress disorder (PTSD), depression, and anxiety [39]. Emergency department crowding is a chronic stress that severely limits capacity in the United States [40] and many other locations globally [41]; events such as large wildfires, increasingly frequent with climate change, drive higher emergency department visit rates for a range of conditions [42] in facilities which are often inadequately prepared for the surge, with quality of care suffering as a result [43].

In response to the limitations of V&A assessments to effectively capture the potential challenges of climate-related shocks and stresses, we propose to supplement these assessments with climate change and health stress tests.

## 4. Conducting a Climate Change and Health Stress Test

Stress testing considers socioeconomic and political factors that can influence the extent of health system vulnerability and factors that can affect demands on the system by impacting population health. A stress test is designed to identify conditions under which it would be difficult for the health system to maintain its essential function of providing services to protect population health and manage climate-sensitive health outcomes, and to identify interventions that could maintain essential system functions despite these shocks and stresses. A stress test focuses on acute and chronic climate-related events and conditions, including those far outside the range of historic experience, that could directly impact health systems and/or climate-related events and conditions in non-health sectors that can indirectly impact health or health system function. This approach can be used to extend the analyses and information gathered during the process of conducting a V&A assessment by leveraging the team, stakeholders, information, and multi-sectoral analyses that ideally accompany a V&A assessment process. The stress test can focus on one or more of the building blocks of health systems, with the aim of improving system resilience, robustness, redundancy, and coordination [7,44]. By doing so, stress testing may both inform ongoing and future V&A assessments, and provide scenario-based analyses that may enhance stakeholder buy-in.

The climate change and health stress test can focus on shocks and/or stresses. The stress test can be approached from the perspective of managing outbreaks of climate-sensitive health outcomes (e.g., Zika, malaria), events that directly affect population health and health system functioning (e.g., extreme weather and climate events such as heatwaves, flooding, storm surges), impacts on other sectors that affect health system function (e.g., cascading systems failures affecting power generation and transmission, hydrometeorological disasters affecting healthcare supply chains), or events from outside health systems that could indirectly affect population health and health system functioning (e.g., large scale migration associated with drought). The following description has a greater focus on shocks for illustrative purposes because these may be the most immediate need for health system officials, but the approach can be modified to address potential climate-related stresses that could compromise health system functioning.

The subsequent sections explain the stages of a climate change and health stress test, followed by a discussion. The activities described in the stages are similar to and draw upon guidance developed for gauging the climate resiliency of health facilities and for conducting V&A assessments [11,19]. Some activities, such as forming a stress test team, reviewing data and information, prioritizing health issues, and communicating results, can be shared between a V&A assessment and a climate and health stress test. The three basic stages in a stress test are as follows:

- *Stage 1: Prepare the climate change and health stress test*. Preparing for a climate change and health stress test includes forming the stress test team; reviewing the available data and information, as well as collecting new data and conducting necessary modeling to inform the scenarios developed; identifying priority health system functions and/or climate-sensitive health outcomes for the stress test; developing hypothetical situations (scenarios) of shocks and stresses that form the core of the stress test; and identifying stakeholders for inclusion in the exercise. Preparing the stress test can benefit from linking with a completed climate change and health V&A assessment or one being conducted in parallel. Ideally, the stress test includes multiple aspects of health systems and services, including elements addressing all six building blocks of climate resilient health services [7].
- *Stage 2: Conduct the stress test.* Various stress testing modalities could be used, alone or in conjunction. At a minimum, table-top or visioning exercises may be completed by the stress test team in a workshop setting, in which the participants discuss the scenarios and evaluate the extent to which health systems would likely be able to manage the shocks and stresses. Additional modalities could be employed, including agent-based and systems dynamics modeling to explore impacts in silico. Regardless of the approach(es) used, the team should review the findings to identify additional resources, tools, and policies that, if made available, could prevent adverse population health consequences associated with the hypothetical situations. Recommended changes in policies and measures should be prioritized to increase the capacity of communities and health systems to prepare for and manage the climate-related shocks and stresses considered.
- *Stage 3: Communicate the results to key stakeholders.* A summary report and other outreach materials should communicate the results to key stakeholders. This report should include an overview of climate and health risks and recommendations for actions, considering factors such as the likelihood and timing of the shocks and stresses, competing demands, windows of opportunity to build resilience based on current and planned projects, and stakeholder concerns and preferences.

## 5. Description of the Stages in a Climate Change and Health Stress Test

### 5.1. Stage 1: Prepare the Study

*Decide the key issues on which to focus*: To begin, the ministry of health, health department, or other health administrative unit engaged with the stress test should decide which aspects of health systems and services to include, such as focusing on the preparedness of facilities and infrastructure, acute care and emergency department capacity, pharmacies, first responders, public health services, and non-governmental organizations, such as the Red Cross/Red Crescent society. These choices will inform decisions about which individuals should comprise the stress test team.

*Form the stress test team*: Ideally, the climate change and health stress test team should include specialists (e.g., public health officials, doctors, nurses) knowledgeable about the health risks of climate change, projections of climate change for the geographic region of interest, health system policies and programs of relevance for climate change and health, policies, and programs in other sectors that could impact population health, local and national plans (e.g., those affecting urbanization), and the local demographics of particularly vulnerable groups. Team members could come from the ministry or department of health, healthcare delivery organizations, universities, non-governmental organizations, and others. As local context is critical, inclusion of participants with intimate knowledge of local health system disaster planning and, crucially, actual disaster experience, is key. Including someone from the local meteorological service would be very helpful to provide insights on the local or regional risks associated with specific types of extreme weather events. Every situation is different, therefore, the team should utilize the specifics of a given context, as well as aim to integrate with existing coordination mechanisms where possible. For example, many countries have a climate change and health technical working group that could be engaged in the process. The team should include the breadth and depth of expertise needed, without becoming too large to be effective. Jurisdictions with specific climate hazards of concern to health systems, for example, mental health impacts in rural, isolated, or northern communities, may require participation of specialists, such as psychologists or social support workers.

Determining the stress test timeframe is key, as the type, magnitude, and frequency of climate-related hazards, as well as the population structure, and issues such as land-use and insurance coverage will vary considerably, depending on the time horizon. Other stipulations, such as the extent of the disaster (which would affect the availability of aid) and impacts on other sectors (e.g., power generation, transport, etc.) should also be determined.

*Conduct a stocktake and review/generate data and information*: In this step, the team gathers data and information to inform the stress test. Stocktaking identifies relevant data, studies, reports, or plans developed by the ministry of health and other organizations. Publications, documents, and reports should be sought that provide details on recent climate-related shocks and stresses, and the effectiveness of health systems in preparing for, and managing, associated morbidity and mortality. For example, in the Vancouver Coastal Health jurisdiction of British Columbia, Canada, a scoping report provided information on climate projections for the region, possible risks that threaten facilities, and recommendations for taking adaptation actions in order to inform efforts to prepare facilities for the impacts of climate change [45]. The stocktake and review should leverage efforts already undertaken, particularly results from health V&A assessments and adaptation planning documents.

Examples of relevant information include (1) trends in temperature, precipitation, and extreme events over recent decades; (2) national or subnational research on the health risks of climate change; (3) national or subnational research on sectors that affect population health, such as the possible impacts of climate change on water and food safety and security; (4) information about the health system (e.g., number and types of specific health workers, number of hospitals and services offered at each, frequency of stockouts, description of supply-chains, baseline health metrics, etc.); (5) information on the spatial distribution of vulnerable groups, particularly those that are likely to need additional assistance or ad hoc services in a disaster, such as those with electricity-dependent life-sustaining devices at home, those on hemodialysis, those on opioid replacement therapy, and pregnant women, among others; (6) information on health- and other insurance coverage, and on the capitalization of the health system, as well as standing plans for rescue support including, but not limited to, mutual aid agreements, emergency, and other sources of funding, and other needed support for disaster recovery; and (7) recent national and subnational climate change and health V&A assessments. Additionally, it may be advantageous to collect other information on physical infrastructure (structure and vulnerabilities of the electricity grid, road networks, etc.) that may be vulnerable during extreme weather events and which affect health systems. Appendix A suggests guiding questions to identify where impacts may occur today or in the future. Additional questions will arise based on specifics of the local context.

Depending on the hypothetical situations (scenarios) of interest, it could be helpful to collect additional data and/or conduct modeling to generate further details for the stress test. For example, if the interest is in extreme events, then it could be informative to model possible increases in the intensity of events in the tail of the distribution in mid-century. The modeling could also consider where and when urbanization could increase, population aging, and other factors that could affect vulnerability to the event.

Although access to data and other resources will improve the stress test, in low-resource situations, it is possible to utilize realistic scenarios (e.g., based on events experienced in similar jurisdictions) in place of those tailored specifically to the jurisdiction in question. In this sense, stress testing both increases the accessibility of climate resilience analysis and may result in the buy-in necessary for senior decision-makers to allocate resources needed for a broader V&A assessment or other forms of analysis.

*Identify priority health system functions to be considered in the stress test*: The stress test team will identify a preliminary list of critical health system functions to consider in the stress test, including measures of effective function, such as minimum number of healthcare professionals, access to essential medical products (including supply chain needs), and financing needed. Health system functions of potential interest include how to manage changes in the magnitude and pattern of climate-sensitive health outcomes over the coming decades with additional climate change, such as increases in the frequency, intensity, and duration of extreme weather and climate events, and the spread of infectious diseases [3]. Informal interviews with ministry of health staff, researchers, and other experts may identify other parameters of potential interest. General metrics of health system competency (e.g., daily all-cause mortality rates, in-hospital mortality rates, and cause-specific morbidity and mortality rates for vulnerable populations) are also important to consider because they provide generalizable indicators that are not specific to particular hazards, and because they capture health effects on populations that are not as directly affected. The stocktake will help with the other steps; for example, information from recent national and local assessments will be useful in identifying priority health system functions for the stress test, considered from the perspective of potential changes in the components of risk: hazards, exposure, and vulnerability. 

*Develop desk-based hypothetical situations (scenarios) of shocks and stresses*: The climate and health stress test team should develop several possible hypothetical scenarios for consideration in the workshop. These can range from relatively simple (e.g., heatwave outside historic experience during which the power grid fails) to more complex scenarios, including, for example, interacting and cascading events (e.g., heatwave coupled with wildfires that significantly affect air quality and also affect agricultural productivity) and/or events that affect supply chains (e.g., flooding that limits the ability to bring in needed supplies of medicines, food, and water, or the ability of affected individuals to reach healthcare). The scenarios could be informed by events that occurred in the past or in other regions. Basing the premise of the scenario in past real-world events, particularly those that took place in similar countries and regions, may help increase engagement from local decision-makers. Scenarios should be tailored to the region of interest, accounting for local factors that could affect the magnitude and pattern of the hazard or response. However, they should also describe threats that are beyond historic experience to account for possible future climate change impacts. The scenarios should test the capacity of health systems to manage shocks and stresses projected with climate change in the coming decades, with lower probabilities but more severe consequences. Real-world events experienced in similar geographic settings and plausible, yet currently infrequent, events, may be used in cases where high-quality data and modelling capacity are low. 

Based on the selection of the aspects of health systems and services of interest in the stress test, the scenarios should incorporate relevant information on the status and location of critical infrastructure (e.g., the probability that the power grid could fail during a heatwave, putting additional people at risk) and other factors that will affect the ability of the health system to manage the situation (e.g., a disease outbreak that significantly increases emergency department visits, increases hospital admission rates, decreases discharge rates, or stresses critical care capacity). A short description of each should be developed for discussion during the climate change and health stress test workshop (stage 2).

*Map stakeholders for inclusion in the stress test workshop*: Strong partnerships, social networks, and inclusiveness are key characteristics of resilience and are important facilitators of effective ad hoc disaster response in the health sector [46]. A climate change and health stress test is an opportunity to identify and strengthen connections among stakeholders. To be effective, the workshop should engage a broad range of stakeholders, from local to national governments and civil society. These stakeholders should include officials in charge of health system entities vulnerable to shocks and stresses, as well as individuals who have decision-making roles in developing and implementing resilience-building strategies, including for upstream drivers of population health. Other potential stakeholders include technical staff at the ministry of health; representatives of healthcare facilities and medical clinics; representatives from the mental health and long-term care sectors, pharmacists, health emergency managers, supply chain workers, public health officials, emergency responders, and others. Additionally, it is generally desirable to have representation from actors serving in national to local organizations and/or government initiatives (e.g., public utilities/service providers, planning agencies, public-private agencies providing household services, etc.); private sector officials (e.g., chambers of commerce, industry groups, etc.); NGOs and community groups; and schools, universities, and research institutes. For health authorities with many pressing priorities, it can be difficult to allocate the time and financial and human resources to undertake a stress test. Including a broad range of stakeholders based on strong social networks and partnerships can help address these challenges.

### 5.2. Stage 2: Conduct the Climate Change and Health Stress Test

The stress test will present and discuss the scenarios, as well as any necessary background information to evaluate how well health systems and services could function during and after the scenario. If other modalities, such as modeling, are used, the findings from these efforts also should be discussed. These discussions should identify what additional resources, tools, and policies could prevent or ameliorate adverse consequences for health system functioning or population health. The discussions could also identify where collaborations could be strengthened, such as among health authorities at different levels of government and among various sectoral partners, for example, the ministry of health and electric and water utilities.

Together, the team conducting the stress test, the ministry/department of health, and other key stakeholders, should draft the agenda and presentations, as well as identify the draft list of participants. Including a relatively large number of participants in the workshop will facilitate information sharing and more diverse discussions. The ministry/department of health and other government partners can be helpful in disseminating invitations to key stakeholders. If particular key stakeholders are not able to attend, then the stress test team should meet with these individuals separately to gather their insights for the exercise.

The stress test workshop length depends on the need to provide participants with detailed background and contextual information on climate change impacts on health and health systems and on the hypothetical scenarios. The goal is to assess the extent to which the assembled actors could manage real events based on the scenarios, and to identify what additional policies, capacity building, and resources would be needed to effectively handle these situations. This is an opportunity for diverse stakeholders to exchange views on risks that could constrain the health system from achieving its goals to promote and protect healthy populations. The discussions can provide insights on what additional information, resources, policies, programs etc. would be needed for the health system to be resilient to the impacts of climate change. For example, the stress test workshop may highlight opportunities for education on key issues of concern, such as the risks of heatwaves on mental wellness, or the long-term psychosocial treatment needs related to trauma from extreme weather events. Appendix B lists possible modules to include in stress test workshops, and Appendix C provides guiding questions to facilitate discussions.

Examples of additional actions for health systems to manage shocks and stresses associated with climate change are presented in Appendix D. There are several factors that participants should consider when recommending which actions to undertake, including:

- Necessity—which actions are most critical;
- Timing—when a particular facility, program or other health system function may be vulnerable to certain climate impacts and when particular actions should be taken in to prepare for and respond to the timing of the hazard and other preparation and response activities;
- Capacity—whether current surge capacity levels and available expertise are sufficient to handle projected impacts, or whether these need to be increased;
- Likely losses—expected increases in morbidity and mortality; facilities where adaptation is prohibitively expensive and should not be undertaken; areas where programs or polices will need to be reevaluated due to unavoidable impacts (e.g., health transportation and supply lines that rely on ice roads); and contingent additional losses if mutual aid is not available); and
- Costs—the economic, political, environmental, and social costs of taking actions to increase resilience, whether they are manageable and how they compare to the costs of inaction.

Prioritizing actions and investments can be done through multi-criteria analyses, or risk-based prioritization. More specific examination of whether the costs of adaptation warrant investment in specific measures may use cost–benefit analyses or cost-effectiveness analyses [19]. The approach used will often depend on the data, expertise, and timeframe available for the analysis and the needs of decision-makers. For example, cost-effectiveness analysis would require robust information on the expected effectiveness of the suite of adaptation options under consideration; this information may be limited, or not available. The recommendations can be categorized into urgent and immediate actions (e.g., capacity building, data collection, records management, social network development, etc.), medium- and longer-term actions (new program development, infrastructure modifications or re-siting, etc.), and measures to strengthen contingency planning for shocks and stresses, thereby reducing the vulnerability of the health system. The recommendations should address specific climate-sensitive health risks, and include measures to protect vulnerable communities, groups, or assets, in the context of low-carbon development.

Stress test participants should attempt to collectively agree on the major findings from the climate change and health stress test, with respect to the ability of different elements of the health system to manage the hypothetical scenarios, as well as additional policies, programs, and actions that could increase their capacity to prepare for and manage the changes. Moreover, there should be discussion of who should follow-up on recommended actions and on next steps for using the results to inform policies to increase resilience to climate variability and change. Participants should also stipulate a timeframe to reassess planning efforts going forward.

### 5.3. Stage 3: Communicate the Results to Decision-Makers and the Public

The climate and health stress test results and recommendations should be communicated to decision-makers in health systems and to other stakeholders through, for example, a summary report and other materials (e.g., policy briefs), taking into consideration the best mechanisms, opportunities, and products to communicate the findings. For example, an investigation of critical public health infrastructure vulnerabilities in California included a map showing the location of hospitals on California’s coast, and current risks associated with a 100-year coastal flood event and future risk from projected sea level rise [47]. Report recommendations should be informed by results from recent climate change and health V&A assessments to provide additional insights into the timing and level of adaptation ambition, taking into account factors such as the likelihood and timing of the threats, competing demands, windows of opportunity based on current and planned projects, and stakeholder concerns and preferences. 

The report also should discuss the limitations of the stress test. The analyzed scenarios are hypothetical situations that, while outside the range of recent experiences, could still underestimate the speed with which vector-borne diseases could change their range or the severity of future extreme weather and climate events, and could overestimate the effectiveness of the health (or other) system responses. The report should indicate how the results could be combined with the findings from other studies, or integrated into future assessments, to maximize a health system unit’s understanding of climate change threats and the opportunities to adapt.

As a component of the iterative management of the health risks of climate change, the stress test should be repeated every few years, if possible, using the previously assessed scenario and updated scenarios, to measure the extent to which preparedness is changing over time, and whether resilience is increasing or decreasing relative to baseline indicators. Authors should discuss the results and how they can be interpreted in the perspective of previous studies and of the working hypotheses. The findings, and their implications, should be discussed in the broadest context possible. Future research directions may also be highlighted.

## 6. Discussion

Even with ongoing mitigation efforts, there is a critical need for health adaptation, as highlighted by realities of locked-in climate change and expected increases in the frequency and intensity of extreme weather and climate events. Health authorities are increasingly responding to that need by taking preventive actions to build climate-resilient health systems (e.g., assessments, global conferences, research studies, partnership building) and the development of decision-support tools to support adaptation (e.g., health facility resiliency tools) [11]. Health authorities also are beginning to monitor progress towards resilience [48]. Climate change and health stress tests build upon these efforts, exploring the extent to which each component of health systems is prepared to manage increases in climate-related shocks and stresses in the context of other challenges—a key indicator of resilience.

Like many of the projected risks of climate change, impacts to health systems will not be felt equally between or within countries. Areas with higher adaptive capacity and lower reliance on external support or resources are likely to be more resilient [3]. Comparatively lower-resourced communities are likely to be affected to a greater extent, and more susceptible to shocks and long-term stresses (e.g., rural and remote health systems). Given the relatively minimal time and resources required to undertake a stress test, it is a technique that is highly accessible to low-capacity health authorities in diverse regional contexts. Information and data provided by an increasing number of climate change and health V&A assessments at national and subnational levels will support robust stress test studies.

As health systems differ significantly within and among countries (e.g., health systems and services serving a remote northern community vs those in a large city with a complex of health centers), the scope and methods employed in stress test studies will need to be tailored to suit the needs of decision-makers. Stress tests provide health authorities with the opportunity to examine the climate resilience of specific components of health systems (e.g., health facilities, specific organizations or departments) or interacting components (e.g., health facilities, integrated disease surveillance and warning, pharmacies, community care, health insurance services). For this reason, the tool can be used by decision-makers responsible for broad health system functions (e.g., a national ministry of health) or specific components (e.g., a health facility). Health authorities conducting stress tests will benefit through sharing of information about experiences in conducting workshops of different scope, in different jurisdictions, and with different partners.

Stress testing utilizes tools familiar to key stakeholders that facilitate vulnerability assessments and similar to methods employed by public health officials, risk-assessors, emergency managers, and decision-makers. In many regions, scenario-based emergency management exercises are mandatory components of all-hazard risk planning. Introducing or augmenting existing activities with climate stress testing would add value to these efforts, and would enhance preparedness planning for a climactically different and potentially more dangerous future. Stress testing should be part of efforts to identify evolving hazards to health systems and to identify and address weak components, feeding into ongoing all-hazards planning.

## 7. Conclusions

Climate change is increasing risks to the health and well-being of people in all countries. According to the Intergovernmental Panel on Climate Change, health impacts are expected to increase with further warming, putting millions more people at increased risk from heatwaves, food insecurity, infectious diseases, water and air pollution, and poverty [49]. Tipping points in climate and social systems that could result in very severe impacts on health are possible. Health services are a first defense in preventing adverse health outcomes from climate change, and are critical for protecting people by accurately diagnosing and treating illnesses and injuries when climate hazards strike. They provide the foundation for individual and community level resilience to climate change.

The World Health Organization has called on health authorities to prepare for climate change risks by building climate resilient health systems, with the urgency of such action increasing as the climate continues to warm. Climate change and health stress testing supports strengthening health systems by assessing and promoting effective and iterative risk management, while fostering engagement across health departments, other sectors, and budgetary authorities. Stress tests can identify actions and investments over the short- to longer-term, to increase the resilience of health systems using a proactive, cost-efficient approach in the context of the upstream determinants of effective health system functioning.

## Appendix A. Guiding Questions for Stage 1

Current burden of climate-sensitive health outcomes	- Are temperature and precipitation associated with the health outcome(s) or with the transmission cycle(s) of interest in the area of interest? How important are the associations to the current burdens of climate-sensitive health outcomes? - Is there evidence that infectious diseases have already changed their geographic range or seasonality of transmission? - Do the number of cases of health outcomes of interest increase or decrease during heatwaves, floods, droughts, and/or storm surges, or have these events affected the transmission cycle? How important is the change? - Are there trends for the hazards, suggesting how climate change could affect the burden of climate-sensitive health outcomes over the shorter term? - Which populations and regions are particularly vulnerable?
Future burden of climate-sensitive health risks	- How much might increasing temperatures and changing precipitation patterns affect the magnitude and pattern of future burdens of the health outcome(s) of interest? How important could this change be over the next few decades? - Could climate change alter the geographic range, seasonality, or intensity of transmission of Lyme disease, dengue, and other infectious and re-emerging diseases? Over what time period? - How could changes in the patterns of extreme events (heatwaves, droughts, floods, extreme storms) affect the burden of climate-sensitive health outcomes? Over what time period? - Based on projections, are health outcomes likely to become more or less sensitive to climate change over time? Could climate change facilitate the emergence of health outcomes that may need more attention? How could hotspots of health outcomes change with climate change?
Environmental factors that could affect population health, including - Availability of safe water (quality and quantity) - Food security - Wildfires - Coastal erosion - Saltwater intrusion	- To what extent do temperature, precipitation, other weather variables, and sea level rise affect access to environmental services? How important is the association? - How could water supply and sanitation systems be affected by changes in precipitation or salinization? - How could wildfires and coastal erosion, for example, affect access to environmental services or electricity networks? - To what extent could changes in these environmental factors affect population vulnerability to the risks associated with climate change?
Social and economic context, including - Community services - Livelihoods - Social capital - Economic resources - Infrastructure	- To what extent do temperature, precipitation, other weather variables, and sea level rise affect social and economic factors? How important is the association? - How could climate change affect the future availability and level of community services? Over what time period? - To what extent could changes in these socioeconomic factors affect population vulnerability to the other risks associated with climate change? Over what time period?
Health systems, including factors such as - Ability to deliver services - Access to healthcare facilities - Supply chains, particularly during extreme weather and climate events	- To what extent do temperature, precipitation, other weather variables, and sea level rise affect the ability of health systems to deliver services? - Have extreme weather and climate events affected access to healthcare systems? How important was the disruption? - Have extreme weather and climate events affected supply chains? How important was the disruption? - Are mechanisms in place to mobilize resources (financial and human) during and after extreme weather and climate events?
Geography and climate	- Which regions could be affected by the climate-related hazard? Over what time period? - Where are the most vulnerable populations for the health outcome(s) of interest located? - Which ecosystems and other services are of importance? Over what time period? - Are there other factors of concern?

## Appendix B. Possible Modules to Include in the Climate and Health Stress Test

- An introduction explaining the broader context of the workshop, and demonstrating the commitment of the ministry/department of health to the stress testing
- Review of the ministry/department of health’s long-term goals and relevant plans
- Background information relevant to the health risks of climate change
- Review of vulnerability, adaptation, and capacity assessments, and of adaptation and mitigation plans
- Introduction to the climate change and health stress test process and outcomes
- Facilitated breakout group discussions on the extent to which the health system could manage the hypothetical scenarios, such as changes in the geographic range, seasonality, or intensity of transmission of climate-sensitive health risks with additional climate change, or changes in the intensity of extreme weather and climate events
- Group discussions on what actions and investments are needed in addition to current policies and programs to reduce and manage increases in climate-sensitive health risks
- Conclusions and next steps

## Appendix C. Guiding Questions for Discussions at a Climate Change and Health Stress Test Workshop

- What is the level of effectiveness of current control programs to manage the burden of disease? How likely could the programs adjust to manage changes in the geographic range, seasonality, and intensity of transmission of, for example, infectious diseases? Or, how quickly could delivery of health services and supply chains be established in case of an extreme weather and climate event outside of historic experience?
- Are there monitoring and surveillance systems that can provide place-based and timely information? An example is collection and analysis of environmental data that could warn when flooding events are expected, along with data on socioeconomic conditions so that vulnerable regions and populations can be identified.
- Are monitoring and surveillance data incorporated into strategic resource planning (financial, infrastructure, medical personnel and training), distribution chains, disaster preparedness, etc.? Does strategic planning consider climate change-related risks and their potential consequences?
- Are memorandums of understanding in place with other ministries and departments to facilitate timely access to data and information?
- Does experience suggest the level of social capital in the community? Are there activities underway in the community that could be extended to increase social capital?
- Has there been an evaluation of climate-related risks to healthcare infrastructure, and of any challenges to maintaining services in cases of epidemics or extreme weather and climate events? If so, what were the implications of this evaluation?
- Are there education and training programs that could easily incorporate the health risks of climate variability and change?

## Appendix D. Examples of Additional Actions for Health Systems to Manage Shocks and Stresses Associated with Climate Change

- Ensure incorporation of health risks into adaptation planning, to strengthen coordination and collaboration across sectors
- Strengthen surveillance, monitoring, and control programs to prepare for climate change-related changes in the geographic range or seasonality of a disease
- Develop early warning and response systems using environmental information to warn of likely outbreaks
- Provide training and capacity building for healthcare professionals to better manage health burdens
- Project how climate-sensitive health outcomes or other risks (e.g., extreme weather and climate events) could change under different scenarios of climate change and development at time periods of interest (e.g., 2030s)
- Consider how development choices could affect future health burdens, for example, the potential consequences of planned urbanization for population vulnerability to flooding
- Improve strategic planning and coordination of policies and programs across departments
- Develop memorandums of understanding and collaborations with other organizations to ensure timely access to and sharing of information and data
- Support research to fill key knowledge gaps
